# Repeated measures of inflammation, blood pressure, and heart rate variability associated with traffic exposures in healthy adults

**DOI:** 10.1186/s12940-015-0049-0

**Published:** 2015-08-15

**Authors:** Jaime E. Mirowsky, Richard E. Peltier, Morton Lippmann, George Thurston, Lung-Chi Chen, Lucas Neas, David Diaz-Sanchez, Robert Laumbach, Jacqueline D. Carter, Terry Gordon

**Affiliations:** Department of Environmental Medicine, New York University, Tuxedo, NY USA; Division of Environmental Health Science, University of Massachusetts, Amherst, MA USA; U.S. EPA; Epidemiology Branch, Chapel Hill, NC USA; U.S. EPA; Environmental Public Health Division, Chapel Hill, NC USA; Department of Environmental and Occupational Medicine, University of Medicine and Dentistry of New Jersey, Piscataway, NJ USA

**Keywords:** Air pollution, Health effects, Traffic, Biomarkers

## Abstract

**Background:**

Previous human exposure studies of traffic-related air pollutants have demonstrated adverse health effects in human populations by comparing areas of high and low traffic, but few studies have utilized microenvironmental monitoring of pollutants at multiple traffic locations while looking at a vast array of health endpoints in the same population. We evaluated inflammatory markers, heart rate variability (HRV), blood pressure, exhaled nitric oxide, and lung function in healthy participants after exposures to varying mixtures of traffic pollutants.

**Methods:**

A repeated-measures, crossover study design was used in which 23 healthy, non-smoking adults had clinical cardiopulmonary and systemic inflammatory measurements taken prior to, immediately after, and 24 hours after intermittent walking for two hours in the summer months along three diverse roadways having unique emission characteristics. Measurements of PM_2.5_, PM_10_, black carbon (BC), elemental carbon (EC), and organic carbon (OC) were collected. Mixed effect models were used to assess changes in health effects associated with these specific pollutant classes.

**Results:**

Minimal associations were observed with lung function measurements and the pollutants measured. Small decreases in BP measurements and rMSSD, and increases in IL-1β and the low frequency to high frequency ratio measured in HRV, were observed with increasing concentrations of PM_2.5_ EC.

**Conclusions:**

Small, acute changes in cardiovascular and inflammation-related effects of microenvironmental exposures to traffic-related air pollution were observed in a group of healthy young adults. The associations were most profound with the diesel-source EC.

**Electronic supplementary material:**

The online version of this article (doi:10.1186/s12940-015-0049-0) contains supplementary material, which is available to authorized users.

## Background

Due to population growth, the expansion of metropolitan areas, and the increasing dependence upon motor vehicles, the fraction of the population residing or working near major roadways has increased, representing a potentially vulnerable population of individuals experiencing adverse health effects related to the inhalation of traffic-related air pollution. In epidemiological studies, exposures to traffic pollutants have been associated with acute and chronic respiratory morbidity [1–3], cardiovascular morbidity [4, 5], and all-cause mortality [6, 7]. In order to better understand the underlying mechanisms involved in these associations researchers have started conducting field panel studies that can allow for more extensive biological monitoring and improved pollutant characterization (i.e., personal or microenvironmental monitoring) to assess the source-related air pollution components responsible for any measured adverse health effects. The results of many field panel studies have uncovered associations between specific traffic pollutants and changes in blood pressure (BP) [8], heart rate variability (HRV) [9], lung function [10], and vascular function [11]; however, many studies have also either failed to find associations with these health effects or found only minor associations [12, 13]. This could be due to differences in the population demographics and/or measured pollutant components across studies. Thus, we undertook a new study in which we conducted detailed exposure assessment monitoring as well as a vast array of health endpoints measured in the same population during exposures to different complex air pollution mixtures.

Recent improvements in technology have allowed researchers to collect personal exposures of air pollutants more easily in a field setting. However, due to a lack of access to an electricity supply, or a place safe enough to carry out medical procedures, it remains difficult to collect as many health measurements in the field compared to a clinical setting. This inability to collect certain health measurements in an ambient setting hampers the ability of researchers to measure transient health effects following pollutant exposures. In this work, we addressed this limitation by using a novel application of dried blood spots (DBS) to gather blood from subjects in the field. This method is minimally invasive, easily performed in the field, requires little previous training to administer, and samples can be collected quickly and repetitively [14–16]. By relying on DBS, the present study extends the previous literature of measuring short-term adverse health effects associated with microenvironmental exposures to traffic-related air pollutants.

We measured a variety of health-related effects in adults before and after walking alongside three public roadways with diverse vehicle types and traffic volumes, providing a more complete range of pollutant concentrations compared to past studies. The health measurements studied in this work have previously been used to demonstrate negative impacts of exposures to traffic pollutants and span multiple biological systems (i.e., cardiovascular, respiratory, and immune), providing a large overview of how toxic pollutants may elicit subclinical health effects in a human population. We used DBS to safely collect blood samples from our subjects away from a medical office to look at acute and lasting changes in blood cytokines related to inflammation. We hypothesized that acute exposure to traffic pollutants would induce mild changes in our health measurements in our healthy human cohort.

## Methods

### Study participants

During the months of June through September of 2011 and 2012, healthy adult participants, aged 18–40 years, were recruited from the Northern New Jersey - Southern New York area via personal contacts and flyers. To minimize risks associated with mild exercise and/or air pollution exposure, participants were excluded if they: 1) did not think they could maintain the required exercise level and duration; and 2) reported tobacco use, asthma or other respiratory disease, type 2 diabetes, or cardiovascular disease. Participants were asked to refrain from consuming caffeine after midnight prior to all exposures. All participants provided written consent, approved by New York University’s School of Medicine’s Institutional Review Board, prior to enrollment.

### Study design

We conducted a randomized crossover study of similar exposure sessions at three locations that were in proximity to roadways with different traffic types but similarly affected by the long- range transport of regional air pollutants. Participants met an investigator prior to each exposure at a designated area within walking distance from the pre-selected exposure roadways for pre-exposure measurements (i.e., a different field setting was used for each location). Once the pre-exposure data collection was completed, each participant walked at a moderate pace (approximately 3 mph) for approximately 2 h on nearly level ground, alternating between 20 min of walking and 5 min of rest. At each location, a pedometer was worn by the study investigator.

Microenvironmental pollution measurements were collected while participants were walking. Immediately and 24 h following each exposure, the participants underwent another series of health measurements. Post-exposure health measurements were collected at the same designated field settings as the pre-exposure measurements. Twenty-four hours after each exposure, health measurements were taken at a field location near the home or workplace of each subject to minimize additional traffic exposures to each subject. All exposure sessions were separated by at least 2 weeks, and were generally confined to the same weekday; participants were permitted to substitute among Tuesday, Wednesday, and Thursday exposures. Between 1 and 5 participants completed each exposure session simultaneously.

### Study locations

The locations chosen for this study included the George Washington Bridge (GWB), the Garden State Parkway (GSP), and a lightly travelled rural road in Sterling Forest (SF), which straddles the border between NJ and NY. The upper deck of the George Washington Bridge connects Northern New Jersey and Northern Manhattan, and has eight traffic lanes open to trucks, buses, and automobiles. A pedestrian walkway is located directly adjacent to the southern side of the upper deck. The GSP is restricted to only automobiles, limiting exposure to diesel engine exhaust. At this location, a sidewalk in a residential area located 30 m from the roadway in Nutley, New Jersey was used. The third site lies in the heart of Sterling Forest State Park in New York and has limited traffic.

### Exposure assessment

Particulate matter (PM) samples were collected using a custom-built mobile sampling platform [17] that collected samples alongside the subjects during their exposures. PM_10_ was sampled using an Aerotec-2 cyclone as a pre-collector of oversized particles at a total flow rate of 171 l per minute (LPM) [18], which was divided between a 37 mm Teflon filter (Pall Corporation, Ann Arbor, MI) and 37 mm quartz filter (Pall). Two additional inlet cyclones limited the sampling to PM_2.5_ at 25 LPM [18], also divided between the 2 filter types. Teflon filters were conditioned and weighed (XS105, Mettler-Toledo Inc., Highstown, NJ) in an environmentally controlled weighing facility for a minimum of 24 h before and after use. Elemental carbon (EC) and organic carbon (OC) were analyzed according to NIOSH Method 5040 (Lab ECOC Aerosol Analyzer, Sunset Instruments Inc., Hillsborough, NC).

A continuously-monitoring, real-time micro-Aethalometer (microAeth model AE51, Magee Scientific, Berkeley, CA) measured black carbon (BC) every minute at a flow of 150 mL/min. A continuous gas analyzer logged measurements of carbon monoxide (CO), nitric oxide (NO), nitrogen dioxide (NO_2_), sulfur dioxide (SO_2_), and ozone (O_3_) concentrations (Advanced Sense Analyzer, GrayWolf Sensing Solutions, Shelton, CT) at 1-min intervals.

Ambient hourly O_3_ concentrations were obtained from the United States Environmental Protection Agency’s Air Quality System Data Mart (http://www.epa.gov/airquality/airdata/). For each location, the closest central monitor collecting O_3_ concentrations was determined, and the selected central monitor was located no further than 10 miles away from each location. Average O_3_ concentrations were calculated between 7 am and 12 pm the day of each exposure.

Hourly relative humidity (RH) and ambient temperature measurements were obtained from the New Jersey Department of Environmental Protection’s Air Quality Index Now reports for the East Orange location (http://www.njaqinow.net/Default.ltr.aspx). Noise measurements were taken at the various locations for a select number of exposures in the summer of 2012. At SF, 1 measurement was taken, while 2 measurements were taken at the GSP and 4 measurements were taken at the GWB. For these exposures, A-weighted noise levels were measured during the four 5 min rest periods and averaged (Radioshack, Sound Level Meter). A-weighted noise levels, as suggested by the Occupational Safety and Health Administration, relates to the human perception of loudness by the human ear and is measured on a logarithmic scale [19].

Traffic volume data for the GSP were obtained for the Essex Toll Plaza using the New Jersey Turnpike Authority’s Open Public Records Act. Traffic volume data at the GWB were obtained for the GWB upper level and Palisades Interstate Parkway tolls from the Port Authority of New York and New Jersey (PANYNJ). Virtually no traffic was observed at SF, as verified by study staff during exposures.

### Health measurements

Health measurements were collected before, immediately after, and 24 h following each exposure. The order of the endpoints (exhaled nitric oxide (eNO), HRV, lung function, BP, and blood prick) was kept consistent.

eNO was measured using a NIOX MINO (Aerocrine AB, Stockholm, Sweden) according to American Thoracic Society’s (ATS) recommendations [20], with the exception that a single measurement was taken at each time point. Lung function parameters (forced vital capacity [FVC] and forced expiratory volume in the first second [FEV_1_]) were measured using a portable spirometer (KoKo Legend, Ferraris Respiratory, Colorado) according to ATS criteria [21]; the subjects were seated, did not wear a nose clip, and the same investigator provided all coaching. Once 3 acceptable readings were recorded, the maximum values were used for analysis.

A digital electrocardiogram (ECG) recording of each subject was acquired continuously for 24 h using a 3-lead Holter monitor (Cardio Data Systems, East Syracuse, NY). At each of the 3 measurement time points, the participant rested for 15 min, and the last 5 min were used for the analysis (Impressario, Version 3.07.0158, Spacelab, Washington). The ECG tracings were manually inspected to correct for mislabeled beats. Time- and frequency-domain indices of the standard deviation of the NN intervals (SDNN), root mean squared of successive deviations in NN intervals (rMSSD), high frequency (HF) (0.15-0.40 Hz), low frequency (LF) (0.04-0.15 Hz), LF:HF, and heart rate (HR) were obtained.

Single systolic BP (SBP) and diastolic (DBP) measurements were obtained using an automated BP monitor (Omron HEM-705CP, Omron Healthcare, Inc, Japan) at each time point. Pulse pressure (PP) was calculated by subtracting the DBP measurements from the SBP measurements. Mean arterial pressure (MAP) was calculated as: $\mathrm{D}\mathrm{B}\mathrm{P}+\frac{1}{3}\left(\mathrm{S}\mathrm{B}\mathrm{P}\hbox{-} \mathrm{D}\mathrm{B}\mathrm{P}\right)$.

Biomarkers of inflammation were measured from capillary whole blood collected on a neonatal Guthrie card (903® Protein Saver Card, Whatman, Westborough, MA). An area of the finger was swabbed with an alcohol pad, dried, and pricked with a lancet. Blood drops were applied to the collection area until full. The DBS were allowed to dry at ambient temperature away from sunlight, and then placed in a gas-impermeable plastic bag and stored at −80 °C. Single measurements were taken for each subject at each time point.

Blood samples were punched from each DBS and placed into individual wells of a 96 deep-well plate (USA Scientific, Ocala, FL). 200 μL of phosphate buffered saline (Sigma-Aldrich, St. Louis, MO) with 0.5 % Tween 20 (Sigma-Aldrich) were added to each well, completely submerging each sample. The plate was covered, cooled, and continuously shaken overnight (Model DS1, IKA Works). The following day, sample eluents were analyzed for protein content (C-reactive protein (CRP), serum amyloid A (SAA), soluble intercellular adhesion molecule (sICAM), soluble vascular adhesion molecule (sVCAM), interleukin 1-beta (IL-1β), interleukin-6 (IL-6), interleukin-8 (IL-8), and tumor necrosis factor-alpha (TNF-α)) using Meso Scale Discovery (SECTOR® Imager 2400, Meso Scale Diagnostics, Gaithersburg, MD). Cortisol was measured using a commercially available kit (R&D Systems, Minneapolis, MN), where absorption was measured with a microplate reader at 450 nm (Ceres UV 900 HDi, Bio-Tek Instruments, Winooski, VT). All samples were run in singlets.

### Statistical analysis

Exposure data were analyzed for differences across locations using an analysis of variance (ANOVA) followed by a Student Newman-Keul’s post hoc test, or, where applicable, an unpaired t-test, in Prism 5.0 for Windows (GraphPad Software, San Diego, CA). Repeated-measures ANOVA was used to assess for statistical differences among pre-exposure health values at the 3 exposure sites. The health outcomes for each observation were then analyzed as a percent change between the pre-exposure measurement and each subsequent measurement (i.e., post or 24 h post) using repeated-measures ANOVA followed by a Student Newman-Keul’s post hoc test. Then, the percent change of health outcomes was associated with each air quality index in a mixed effects model with a random subject intercept, fixed indicators for location, and fixed linear effects for apparent temperature and the exposure of interest using the lme4 package version 1.1-7 in R (R Foundation for Statistical Computing, Vienna, Austria) version 3.1.1. The fixed indicator for location was used to address any possible influences due to the location independent of air pollution. To test whether differences in test location should be included in the model, a separate mixed effect model with location removed, was also run. The results from this model showed decreases in the magnitude of the point estimates, suggesting that differences in location could be influencing the health effects measured. Therefore, we chose to include location in our model to assess changes in health outcomes solely due to the air pollution metrics measured. Apparent temperature was calculated using a formula [22] which takes into account ambient temperature and relative humidity.

## Results

### Participant characteristics

Thirty-six volunteers were recruited for this study; 2 participants were excluded due to smoking status and 8 participants were not available to participate during our time frame. Of the remaining 26 subjects, 21 participants completed 3 exposures, 2 subjects completed 2 exposures, and 3 subjects completed only 1 exposure. The characteristics of the subjects can be found in Table 1, and the pre-exposure values of the biological endpoints are shown in Table 2. Little variability existed among the pre-exposure values at the 3 locations, with the exception of SAA and CRP in which statistically significant pre-exposure values were elevated at GWB compared to SF and GSP (Table 2).

**Table 1:** Subject characteristics

All subjects	23
Gender	
Male	11 (48 %)
Female	12 (52 %)
Age (years)	25 (18–33)
Weight (lbs)	162 (116–270)
Body mass index	25 (20–39)
Race/ethnicity	
White	17 (74 %)
Black	1 (4 %)
Asian	2 (9 %)
Hispanic	2 (9 %)
Other	1 (4 %)
Past smoker	2 (9 %)
Smoker at home	0 (0 %)

Values are represented as either mean (range) or mean (%) for 23 subjects

**Table 2:** Pre-exposure values of biological endpoints

		SF	GSP	GWB	*P*-value
	Subjects (n)	Mean (range)	Mean (range)	Mean (range)	
Respiratory measurements					
eNO (ppb)	19	22 (8–66)	24 (9–61)	19 (9–50)	0.14
FVC (L)	18	4.6 (3.3-6.8)	4.7 (3.2-6.6)	4.6 (3.3-6.5)	0.96
FEV_1_ (L)	18	3.8 (2.7-6.1)	3.7 (2.6-5.7)	3.8 (2.7-5.9)	0.61
FEV_1_/FVC	18	0.8 (0.6-0.9)	0.9 (0.6-1.0)	0.8 (0.6-1.0)	0.44
Inflammatory measurements					
CRP (ng/mL)	17	30.1 (1.3-93.9)	31.6 (0.9-149.3)	53.2 (6.3-225.3)	0.01*
SAA (ng/mL)	16	32.1 (6.1-93.9)	29.8 (3.9-88.3)	66.5 (9.4-355.1)	0.08
sICAM (ng/mL)	17	4.4 (2.2-8.1)	4.5 (1.7-7.3)	5.5 (2.5-8.8)	0.01*
IL-1β (pg/mL)	21	3.7 (0.4-13.9)	3.6 (0.2-17.7)	3.0 (0.2-25.9)	0.86
IL-8 (pg/mL)	21	3.3 (0.8-15.3)	2.4 (0.0-6.7)	2.0 (0.8-5.0)	0.13
Cortisol (ng/mL)	10	0.4 (0.1-0.8)	0.4 (0.2-0.6)	0.3 (0.1-0.6)	0.21
Blood pressure measurements					
SBP (mmHg)	18	126 (106–166)	124 (95–162)	129 (109–155)	0.27
DBP (mmHg)	18	82 (66–103)	82 (64–97)	79 (56–99)	0.21
PP (mmHg)	18	43 (23–70)	41 (19–65)	46 (15–63)	0.14
MAP (mmHg)	18	97 (81–124)	96 (76–119)	96 (78–118)	0.91
Heart rate variability measurements					
SDNN (ms)	18	77 (34–186)	72 (28–164)	75 (43–106)	0.81
rMSSD (ms)	18	41 (15–118)	43 (13–161)	48 (18–94)	0.57
HF (ms^2^)	18	1048 (23–6822)	1054 (20–7261)	1106 (52–4574)	0.99
LF (ms^2^)	18	1651 (407–4835)	2483 (110–13518)	1599 (250–4186)	0.23
LF:HF	18	5.0 (0.5-20.6)	4.9 (0.2-13.1)	3.0 (0.3-6.6)	0.10
Heart rate (BPM)	18	77.8 (58.9-94.9)	78.9 (60.4-118.1)	78.7 (62.2-128.0)	0.94

*SF* Sterling Forest, *GSP* Garden State Parkway, *GWB* George Washington Bridge. Statistically significant differences in pre-exposure values were assessed using repeated-measures ANOVA. *p-value < 0.05

Pre-exposure values expressed as mean (range) at each location

### Exposure assessment measurements

In total, SF was utilized 15 times, GSP 14 times, and GWB 9 times over the 2 summer exposure periods. No statistically significant differences were observed for the driving distance between the exposure sites and the homes of each participant, the walking pace during exposures, and the traffic counts at the GSP and GWB locations (Additional file 1). Noise levels were elevated at GSP and GWB compared to SF, although only a single measurement of noise was taken at SF (Additional file 1).

There was a trend of increasing pollution levels, with SF exhibiting the lowest and GWB exhibiting the highest average concentrations, for PM mass and carbonaceous constituents (Table 3). However, as expected for a regional pollutant, ambient O_3_ concentrations did not vary between locations (p = 0.92). A correlation matrix between the measured pollutants and meteorological conditions is shown in Table 4. PM_2.5_ EC was strongly and positively correlated with PM_10_ EC and BC. PM_2.5_ and PM_10_ were weakly associated with the other pollutants. The mean SO_2_, NO, NO_2_, CO, and O_3_ concentrations collected from the portable instrument were near or below the instrument’s detection limit.

**Table 3:** Pollutant and meteorological concentrations at each location

	Overall means	SF	GSP	GWB	*P*-value
PM_2.5_ (μg/m^3^)	20	13 (7–24)	21 (9–50)	31 (11–45)	< 0.01
PM_10_ (μg/m^3^)	26	16 (6–29)	26 (17–48)	38 (21–50)	< 0.01
PM_2.5_ EC (μg/m^3^)	2.0	0.6 (0.0–1.7)	1.7 (0.1–3.3)	5.3 (0.1–13.2)	< 0.01
PM_10_ EC (μg/m^3^)	2.6	0.9 (0.1–2.5)	2.3 (0.0–7.2)	6.6 (2.2–15.7)	< 0.01
BC (μg/m^3^)	3.3	1.5 (0.2–3.6)	2.8 (1.3–4.1)	7.2 (4.1–10.9)	< 0.01
PM_2.5_ OC (μg/m^3^)	13.9	10 (4–20)	13 (8–20)	21 (12–29)	< 0.01
Ozone (ppm)^a^	0.05	0.04 (0.02–0.06)	0.04 (0.01–0.06)	0.04 (0.02–0.06)	0.92
Temperature (°F)^b^	74	71 (50–81)	75 (66.87)	75 (70–91)	0.24
Relative humidity (%)^b^	72	69 (47–95)	79 (56–97)	65 (49–84)	0.03

*SF* Sterling Forest, *GSP* Garden State Parkway, *GWB* George Washington Bridge. *P*-values were calculated using a 1-factor ANOVA

^a^Hourly ozone concentrations were obtained from the US EPA Air Quality System Data Mart

^b^Hourly relative humidity and ambient temperature measurements were obtained from New Jersey Department of Environmental Protection’s Air Quality Index Now

Values expressed as mean (range)

**Table 4:** Pearson's Correlation Coefficients between air pollutant measurements and meteorological measurements

	PM_2.5_	PM_10_	PM_2.5_ EC	PM_10_ EC	BC	PM_2.5_ OC	O_3_	Temp
PM_2.5_								
PM_10_	0.66							
PM_2.5_ EC	0.29	0.47						
PM_10_ EC	0.23	0.44	0.93					
BC	0.38	0.63	0.88	0.84				
PM_2.5_ OC	0.40	0.59	0.54	0.46	0.60			
O_3_	0.39	0.28	−0.20	−0.31	0.12	0.28		
Temp	0.47	0.50	−0.10	−0.13	0.14	0.25	0.26	
RH	−0.38	−0.08	−0.05	0.00	−0.18	0.13	−0.32	−0.08

*EC* Elemental carbon, *BC* Black carbon, *OC* Organic carbon

Values represent correlation coefficients at all the locations combined

### Health effects associated with air pollutant measurements

**Fig. 1:**
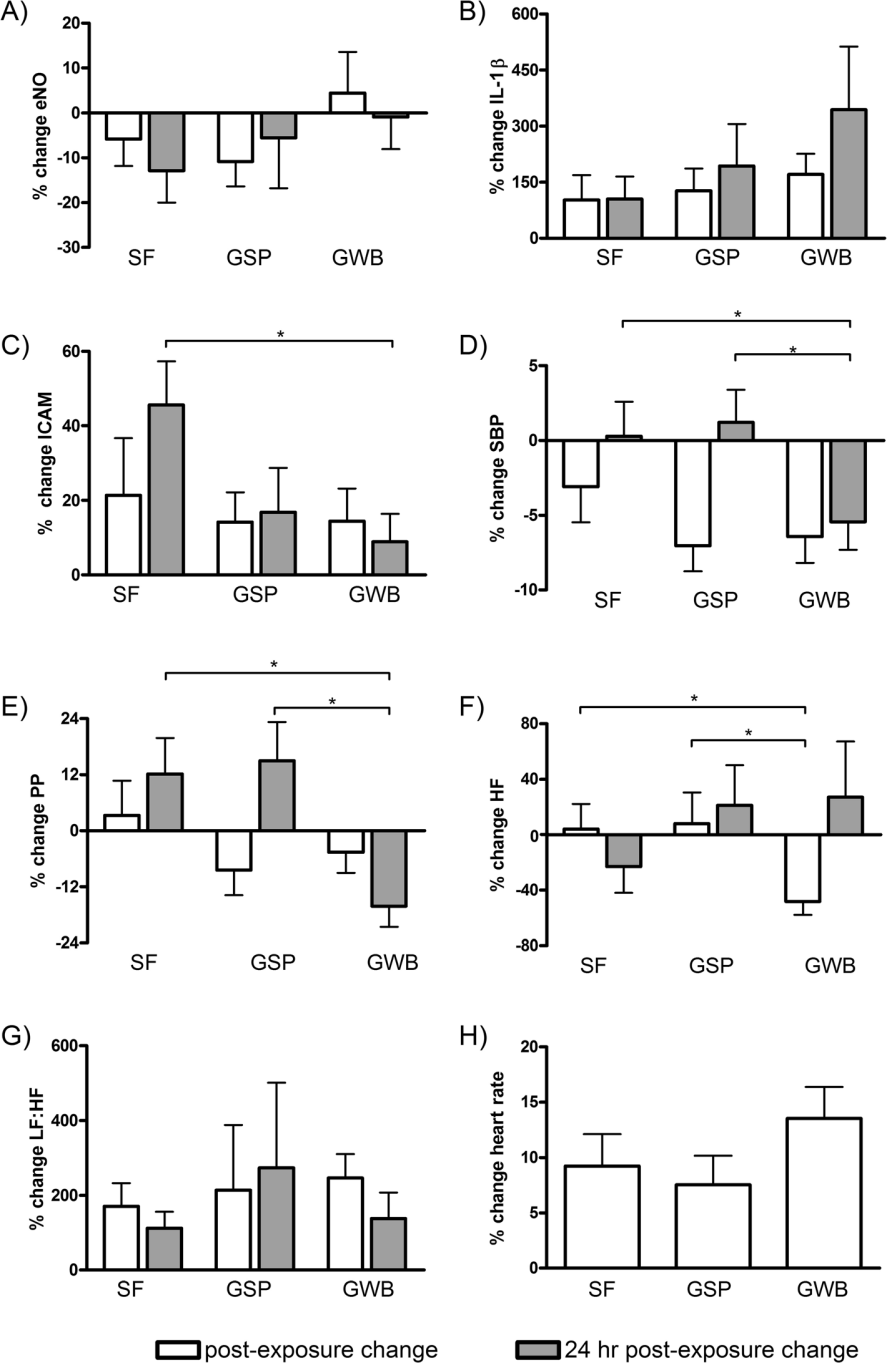
Percent changes in **a** eNO, **b** IL-1 β, **c** ICAM, **d** SBP, **e** PP, **f** HF, **g** LF:HF, **h** HR at SF, GSP, and GWB for post- and 24 h measurements, compared to pre-exposure measurements. Health outcomes were analyzed using a repeated-measures ANOVA to assess statistical significance between locations, followed by a Student Newman-Keul’s post hoc test. Values represent mean ± SE. *p value < 0.05

Health effects were assessed both using a site-by-site comparison and a mixed effect model for pollution and location. When analyzed on a site-by-site basis (Fig. 1), lung function measurements did not differ between locations (Additional file 2). However, an approximate 5 % increase in eNO was observed at the GWB post exposure, whereas all other measurements of eNO were decreased, when compared to pre-exposure values. However, the increase at the GWB was not statistically significant (*p* = 0.13).

Post exposure increases in IL-1β were found at all locations, with the greatest increase at GWB at 24 h post exposure. For sICAM, CRP, and SAA, the greatest percent changes were measured at SF 24 h following exposures. For sICAM the decrease observed at GWB compared to SF was statistically significant. No significant differences in cortisol were found following exposures at any location (Additional file 2). sVCAM, IL-6, and TNF-α values were below the assay detection limits.

When assessing the influence of location on cardiovascular endpoints, decreases in SBP and PP were observed post exposure at SF, GSP, and GWB, and these decreases were sustained at GWB 24 h following exposure. An approximate 5 % decrease in DBP was also observed immediately following all exposures, but these decreases were not statistically significant. Decreases in the HF HRV metric were observed immediately following GWB exposures, and this was significantly reduced compared to the SF and GSP locations. Increases ranging from 10-55 % in the LF HRV metric were found following all exposures, but these data were variable and non-significant. Large increases in LF:HF were observed at all locations both immediately and 24 h following exposures, but these increases were fairly uniform across all locations. No significant changes in heart rate immediately following exposures was observed at any location (Fig. 1).

On a site-by-site basis, it appeared that the GWB elicited the greatest change in the health outcomes measured. Thus, in the mixed effect model the location was added as a fixed indicator to remove any influence of the location on the measured health effects and only used to assess differences in air pollutants. With the exception of a 14.94 % (95 % CI: −0.56, 30.43) increase in blood IL-1β levels, PM_10_ mass concentrations did not greatly (i.e., greater or less than 5 %) contribute to changes in any of the health outcomes measured (Additional file 3). Additionally, as PM_2.5_ EC was strongly and positively correlated with both PM_10_ EC and BC, and the direction and magnitude of changes were similar between the pollutants, the results of associations between health measurements and PM_10_ EC and BC, along with O_3_, can be found in the Supplemental Material (Additional file 3) and the results of the mixed effect model for PM_2.5_, PM_2.5_ EC, and PM_2.5_ OC can be seen in Table 5.

**Table 5:** Slopes (95 % Confidence Intervals) from mixed-effect models^a^ between percent changes in health effects and pollutants

	PM_2.5_	PM_2.5_ EC	PM_2.5_ OC	PM_2.5_	PM_2.5_ EC	PM_2.5_ OC
	Post			24 h		
Respiratory measurements						
eNO	-0.38 (-1.06,0.31)	-1.37 (-3.99.1.25)	-2.08 (-3.70,-0.46)	0.87 (-0.09,1.82)	-0.14 (-4.03,3.74)	-2.14 (-4.49,0.21)
Inflammatory measurements						
CRP	0.32 (-1.37,2.01)	1.17 (-5.42,7.75)	-0.29 (-4.41,3.83)	-1.87 (-4.37,0.64)	-4.97 (-14.82,4.88)	-0.44 (-6.57,5.69)
SAA	-0.29 (-1.57,0.99)	-1.15 (-6.00,3.70)	-0.90 (-4.06,2.26)	-2.59 (-4.53,-0.64)	-8.03 (-15.55,-0.52)	-5.47 (-10.23,-0.72)
sICAM	-0.44 (-1.80,0.92)	-0.98 (-6.06,4.09)	-1.13 (-4.41,2.14)	-1.21 (-2.52,0.09)	-0.74 (-5.85,4.37)	-2.13 (-5.43,1.17)
IL-1β	2.66 (-2.29,7.60)	2.32 (-17.15,21.79)	4.38 (-16.15,7.40)	3.82 (-8.12,15.76)	55.09 (10.91,99.27)	-2.23 (-31.45,26.99)
IL-8	0.37 (-0.77,1.52)	-3.80 (-8.21,0.62)	-1.23 (-3.93,1.48)	0.47 (-1.09,2.02)	-1.26 (-7.45,4.92)	-1.92 (-5.71,1.87)
Cortisol	-0.37 (-2.02,1.28)	-0.35 (-6.88,6.18)	1.94 (-1.85,5.72)	-1.06 (-4.18,2.05)	-12.56 (-25.64,0.51)	4.80 (-2.34,11.94)
Blood pressure measurements						
SBP	-0.04 (-0.31,0.24)	-1.65 (-2.62,-0.68)	-0.06 (-0.72,0.59)	-0.17 (-0.48,0.14)	-1.70 (-2.81,-0.58)	-0.28 (-1.02,0.47)
DBP	-0.11 (-0.41,0.19)	-0.74 (-1.88,0.40)	0.33 (-1.05,0.39)	-0.25 (-0.67,0.18)	-1.02 (-2.65,0.61)	-0.82 (-1.86,0.22)
PP	0.04 (-0.80,0.89)	-4.43 (-7.53,-1.32)	0.28 (-1.73,2.29)	-0.30 (-1.17,0.58)	-3.99 (-7.77,-0.70)	0.69 (-1.40,2.78)
MAP	-0.07 (-0.32,0.17)	-1.11 (-2.00,-0.22)	-0.20 (-0.78,0.38)	-0.20 (-0.50,0.11)	-1.28 (-2.43,-0.14)	-0.56 (-1.31,0.18)
Heart rate variability measurements						
SDNN	0.09 (-0.83,1.01)	0.15 (-3.26,3.57)	0.00 (-2.07,2.07)	-0.39 (-1.28,0.51)	-0.90 (-4.34,2.55)	0.70 (-1.27,2.68)
rMSSD	-0.03 (-0.89,0.82)	-1.86 (-4.99,1.28)	-0.17 (-2.07,1.73)	-0.82 (-1.82,0.18)	-4.35 (-7.89,-0.81)	0.02 (-2.26,2.30)
HF	0.13 (-2.01,2.27)	0.04 (-7.86,7.94)	4.57 (-0.04,9.18)	-0.05 (-3.77,3.67)	-7.57 (-21.92,6.78)	-0.38 (-8.50,7.74)
LF	-0.84 (-4.88,3.20)	-3.22 (-19.10,12.66)	-0.31 (-9.17,8.56)	0.63 (-2.55,3.81)	6.67 (-5.66,18.99)	-0.24 (-7.19,6.71)
LF:HF	2.09 (-11.63,15.81)	24.47 (0.93,48.00)	1.15 (-12.55,14.84)	1.99 (-15.39,19.36)	26.71 (3.82,49.60)	4.73 (-9.13,18.59)
Heart rate	-0.01 (-0.36,0.34)	0.65 (-0.59,1.88)	0.14 (-0.61,0.90)	0.16 (-0.25,0.57)	1.63 (0.25,3.00)	-0.46 (-1.36,0.45)

^a^The mixed effect model adjusted for apparent temperature, location, and random subject effects

Percent change was calculated between the pre-exposure measurement and each subsequent measurement (i.e. post or 24 hrs post)

PM_2.5_, PM_2.5_ EC, and PM_2.5_ OC mass concentrations had very minimal associations with changes in respiratory measurements, and only modest (~2 %) decreases in eNO were observed with increasing levels of PM_2.5_ OC, although this was not statistically significant (Table 5). Therefore, any effect found solely on a site-by-site basis for eNO was lost using our mixed effect model. In addition, minimal changes in CRP, sICAM, and IL-8 were observed, and statistically significant 5-8 % decreases in SAA at 24 h following exposures were observed with increasing PM_2.5_, PM_2.5_ EC, and PM_2.5_ OC concentrations. Increases in IL-1β were found with PM_2.5_, PM_2.5_ EC, and PM_2.5_ OC, consistent with the results obtained looking at changes in IL-1β on a site-by-site basis, and this was significant for PM_2.5_ EC 24 h post-exposure. A trend of decreasing cortisol was observed, but this was not statistically significant.

In looking at the effect of traffic PM components on BP measurements, small negative effects (i.e., changes less than 2 %) were observed for SBP, DBP, and MAP (Table 5), similar to those observed when assessing the results solely based on location (Fig. 1). The largest decreases, ranging between 2.7-3.8 %, were found with PM_2.5_, PM_2.5_ EC, and PM_2.5_ OC measurements and PP 24 h after exposure. When assessing associations between pollutants and measures of HRV, decreases in the root mean squared of successive deviations in NN intervals (rMSSD) were observed, with the greatest reduction (4.4 % decrease) associated with PM_2.5_ EC (Table 5). Increases in HF were found immediately following exposures, yet decreases in HF were observed 24 h after exposures; the magnitude of these changes was minimal with the exception of a 7.6 % decrease with PM_2.5_ EC. The LF:HF was positively associated with the measured pollutants both immediately after and 24 h following exposures.

Sensitivity analyses were conducted by removing 2 subjects with a body mass index (BMI) > 39, 2 subjects with a past history of asthma, and 1 subject that reported anxiety during an exposure. No significant changes in the group mean results were observed with the removal of the past asthmatic and anxious subjects; however, it appeared that the exclusion of the 2 obese subjects reduced the associations observed between PM_2.5_ EC and BP, HRV, and cytokine measurements (Additional file 4).

## Discussion

In the healthy participants in this study, acute exposures to traffic pollutants elicited cardiovascular and inflammatory changes that were most strongly associated with PM_2.5_ EC and OC. This present study is unique in its use of multiple locations and diverse outcomes measured. We also describe a novel application of a blood collection technique suitable for field use. We collected PM and gaseous concentrations using a custom-built, high-volume mobile sampling platform [17]. The design of the platform allowed us to collect sufficient PM to reliably obtain filter weights and EC/OC concentrations in a relatively short sampling period. In this study, we observed elevated PM_2.5_ and PM_10_ at the GWB compared to GSP; the increased number of diesel-fueled engines at the GWB likely contributed to the difference in PM concentrations [23]. We also observed elevated EC, BC, and OC concentrations at GSP compared to SF. Although the GSP cars-only location was picked with the intent of being far from major roadways carrying diesel-fueled vehicles, reports have suggested that the contribution of BC from gasoline exhaust may be underestimated [24].

In addition to PM, a small group of traffic-associated gases were measured during exposures; however, these concentrations rarely exceeded the limit of detection, and could not be used reliably for analysis. Regional O_3_, however, was included in the model and was not associated with any measured health outcomes as part of this work. As this work focused on microenvironmental exposures to traffic pollutants, ambient NO_2_ concentrations measured at central monitors were not explored, particularly as NO_2_ has been shown to be elevated near roadways and then decline to background levels after 100–500 m [25, 26]. However, we cannot rule out the possibility that changes in health measurements could be associated, in part, to NO_2_ or other gaseous pollutants.

In the mixed effects model, we found slight decreases in eNO with increasing concentrations of PM_2.5_ OC and EC. However, when eNO was analyzed on a site-by-site basis, eNO decreased at GSP and SF, whereas a 5 % increase was found at the GWB immediately following exposures. Although this increase in eNO at the GWB was not statistically significant, in previous studies, such increases in eNO have been associated with benzene [27], particle number [28], soot [28], ultrafine particles [29], and carbon monoxide [30].

Various hypotheses have been proposed to explain the interaction between exposure to PM and systemic inflammation. One hypothesis suggests that inhaled PM can induce a local inflammatory response in the pulmonary system. According to another hypothesis, PM penetrates into the vascular system directly, interacting with endothelial cells that release cytokines into the blood [31, 32]. It has also been speculated that airway injury due to the inhalation of air pollutants can increase levels of IL-6 and IL-8, activating mononuclear and endothelial cells which further initiate the secretion of acute-phase proteins and cellular adhesion molecules [33]. In testing these ideas, we did not detect, with the exception of statistically significant increased levels of IL-1β, systemic inflammatory responses. A few studies have assessed IL-1β in the blood of human participants following ambient pollution exposures, and found increases in IL-1β associated with PM_10_ [31], ambient air pollution [34], and diesel [32]. For the remaining serum proteins, results were inconsistent among studies as to whether increases or decreases in proteins were observed [34–39]. These inconsistent results could be related to the study populations or the time points used.

In the current study we used a novel application of DBS to assess changes in pro-inflammatory genes in a field setting. There are several advantages to this technique. First, the use of DBS decreases the risks associated with the use and disposal of needles and syringes for the collector, as well as only requiring a small amount of blood for the analysis, making it attractive to the subject as well [15, 40]. Further, past work looking at the utility of DBS has found the same level of precision and reproducibility as vacuum tubes and capillary pipettes [16], and DBS are considered a Food and Drug Administration registered *in vitro* class 11 medical device. Due to its utility and relatively inexpensive nature for large epidemiological studies, DBS analysis has already been used for measuring therapeutic drugs and drugs of abuse, management of viral disease, quantification of environmental contaminants (i.e. trace metals), and neonatal screening programs [41–45]. Therefore, we believe that this technique could be of high value to future researchers involved in field studies that still wish to look at biomarkers in the blood of their subjects.

One of the interesting findings related to this work included small but negative associations between traffic-related air pollutants and measurements of BP. This was in contrast to the results presented in many previously conducted studies [46–48]. However, decreases in BP measurements were observed both in the site-by-site comparison as well as the mixed effect model analyses. For the HRV analysis, our findings were consistent with past studies that identified associations between increases in LF:HF, and the inhalation of EC [49] and BC [50]. It is interesting that large and statistically significant positive associations were found with LF:HF, particularly with PM_2.5_ EC, although small and sometimes negative associations were found for LF and HF separately in our study. It should be noted that the range in the LF and HF measurements was very large, which could contribute to this discrepancy. It is also interesting that the LF:HF, which has generally been associated with sympathetic modulation, was found to be increased yet BP measurements were decreased after exposure. According to Billman, the LF:HF can vary based on factors other than sympathetic nerve activation, such as the mechanical effects of respiration and prevailing heart rate. Additionally, the sympathetic and parasympathetic nervous system are not linearly related, which could disrupt their balance. Therefore, LF:HF might not solely reflect sympatho-vagal balance, which could account for an increase in LF:HF yet a decrease in BP [51]. In our work, decreases in rMSSD were observed 24 h following exposures, and these decreases were associated with increasing PM_2.5_ EC. rMSSD is a short-term measure of vagal tone [52], and the observed decrease is consistent with other published work of EC [53], OC [53], and PM_2.5_ [50, 54]. Thus, the current study supports results in the literature regarding traffic-related pollutants and their impact on HRV endpoints, but the BP measurements found in this study are in contrast to previously published work.

In carrying out this study, it became apparent that the GWB location could have contributed to additional stress leading to adverse cardiovascular events, particularly from higher levels of noise [55, 56]. The noise levels were elevated at the GWB and GSP locations compared to SF, but these noise measurements were limited and we therefore did not control for noise in our models or perform statistical tests. We did, however, control for location in our model, which should have alleviated any noise-related influences between locations. Other investigators have suggested that cortisol could serve as an adequate marker of noise-related stress [57], and therefore we measured cortisol levels in peripheral blood. In the study cohort from the first summer, no significant differences in cortisol levels were found among the 3 study sites; due to blood sample limitations, cortisol concentrations were not measured from the participants for the second summer.

In the current study, multiple sensitivity analyses were required, especially with a relatively small sample size. It appeared that the exclusion of subjects that were anxious or had a previous history of asthma did not have a large impact on our results, whereas the exclusion of 2 obese subjects affected many of the observed effects, suggesting that subjects with an elevated BMI might be able to modulate the cardiovascular and immune responses. Although the number of obese subjects in this study was small, a recent review puts forth the notion that obesity can influence susceptibility to air pollution-induced cardiovascular health effects [58].

Several limitations to this work exist. First, although we calculated the distance each subject traveled to each location and found no significant differences, we were unable to control for ambient exposures during transport to study sites or for any possible additional pollutant exposures between the post- and 24 h-health measurements. However, pre-exposure biological measurements for each subject were taken at each location, which should have controlled for any pre-exposure effects, and 24 h measurements were taken near the home or workplace of each subject in an attempt to alleviate additional traffic exposures. Also, in this work we did not determine exercise load or ventilation rates and therefore could not calculate the dose of pollutants each participant inhaled. Locations, however, were selected that were on fairly level ground to prevent changes in exertion, the walking pace was controlled with no significant difference found between locations (Additional file 1), and we measured the HR of each subject before and after each exposure and observed minimal changes. In this work we also observed a large variability in the pre-exposure protein concentrations between subjects, which could be a result of obtaining only singlet samples at each time point; as we still found significant inductions, we assume the singlet measurements were adequate. It is also possible that the small sample size limited our ability to see changes in the selected health endpoints. Lastly, it is possible that, due to the large number of statistical tests run, some statistically significant associations may have occurred by chance.

Strengths of this study include microenvironmental monitoring of air pollutants, which has proven to be optimal for studying pollutant-induced health effects, thereby limiting exposure measurement error. The novel application of DBS to air pollution field studies, which are easy to collect, minimally invasive, easy to transport, and useful for sampling in remote areas shows great potential for future work. Lastly, the use of a randomized crossover study design allowed for each subject to act as their own control thereby reducing within-subject covariates.

## Conclusions

In conclusion, we have shown that in our study population of 23 subjects, acute physiological and biological changes can occur in a healthy population following a 2 h walking exposure to near-walkway traffic. Associations were found between increases in markers of inflammation in the blood and decreases in heart rate variability with increasing concentrations of EC. Overall, acute changes in cardiovascular measurements and markers of inflammation in the blood were observed in healthy adults exposed to traffic-related pollution.

## Additional files

Additional file 1:
**Additionally measured exposure metrics at each location.** Values represent means ± SD. Traffic volume data for the GSP were obtained for the Essex Toll Plaza using the New Jersey Turnpike Authority’s Open Public Records Act. Traffic volume data at the GWB were obtained for the GWB upper level and Palisades Interstate Parkway toll records from the PANYNJ. No traffic was observed at SF, as verified by study staff during exposures. SF = Sterling Forest; GSP = Garden State Parkway; GWB = George Washington Bridge. N/A = not applicable. ^a^Unpaired t-test. ^b^Only a single measurement of noise was made at SF. Noise levels at SF without sampler on were below detection limit for instrument (dB < 60). Additional file 2:
**Percent changes in A) FVC, B) FEV**_**1**_ **, C) FEV**_**1**_ **/FVC, D) CRP, E) SAA, F) IL-8, G) DBP, H) MAP, I) SDNN, J) rMSSD, K) LF, and L) cortisol at SF, GSP, and GWB for post- and 24 h measurements, compared to pre-exposure measurements.** Health outcomes were analyzed using a repeated-measures ANOVA to assess statistical significance between locations, followed by a Student Newman-Keul’s post hoc test. Values represent mean ± SE. **p* value < 0.05. Additional file 3:
**Slopes (95 % Confidence Intervals) generated from mixed effect models between percent changes in pollutant concentrations and health effects for the subjects, adjusting for apparent temperature, location, and random subject effects.** Percent change were calculated between the pre-exposure measurement and each subsequent measurement (i.e. post or 24 h post). Additional file 4:
**Slopes (95 % Confidence Intervals) generated from mixed effect models between percent changes in pollutant concentrations and health effects for the subjects, adjusting for apparent temperature, location, and random subject effects.** Analysis was completed removing 2 subject that presented with BMI > 39. Percent change were calculated between the pre-exposure measurement and each subsequent measurement (i.e. post or 24 h post).

## Abbreviations

ANOVA: Analysis of variance
ATS: American Thoracic Society
BP: Blood pressure
CO: Carbon monoxide
CRP: C-reactive protein
DBP: Diastolic blood pressure
DBS: Dried blood spots
EC: Elemental carbon
ECG: Electrocardiogram
eNO: exhaled nitric oxide
FEV_1_: Forced expiratory volume in the first second
FVC: Forced vital capacity
GSP: Garden State Parkway
GWB: George Washington Bridge
HF: High frequency
HR: Heart rate
HRV: Heart rate variability
IL-1β: Interleukin 1-beta
IL-6: Interleukin-6
IL-8: Interleukin-8
LF: Low frequency
LPM: Liters per minute
MAP: Mean arterial pressure
NO: Nitric oxide
NO_2_: Nitrogen dioxide
O_3_: Ozone
OC: Organic carbon
PANYNJ: Port Authority of New York and New Jersey
PM: Particulate matter
PP: Pulse pressure
RH: Relative humidity
rMSSD: root mean squared of successive deviations in NN intervals
SAA: Serum amyloid A
SBP: Systolic blood pressure
SDNN: Standard deviation of the NN intervals
SF: Sterling Forest
sICAM: Intercellular adhesion molecule
sVCAM: Vascular adhesion molecule
SO_2_: Sulfur dioxide
TNF-α: Tumor necrosis factor-alpha
