# Supplementary figures and images for: Repeated measures of inflammation, blood pressure, and heart rate variability associated with traffic exposures in healthy adults
Source: Environ Health. 2015 Aug 15;14:66. doi: 10.1186/s12940-015-0049-0 (PMC4537534; doi:10.1186/s12940-015-0049-0)

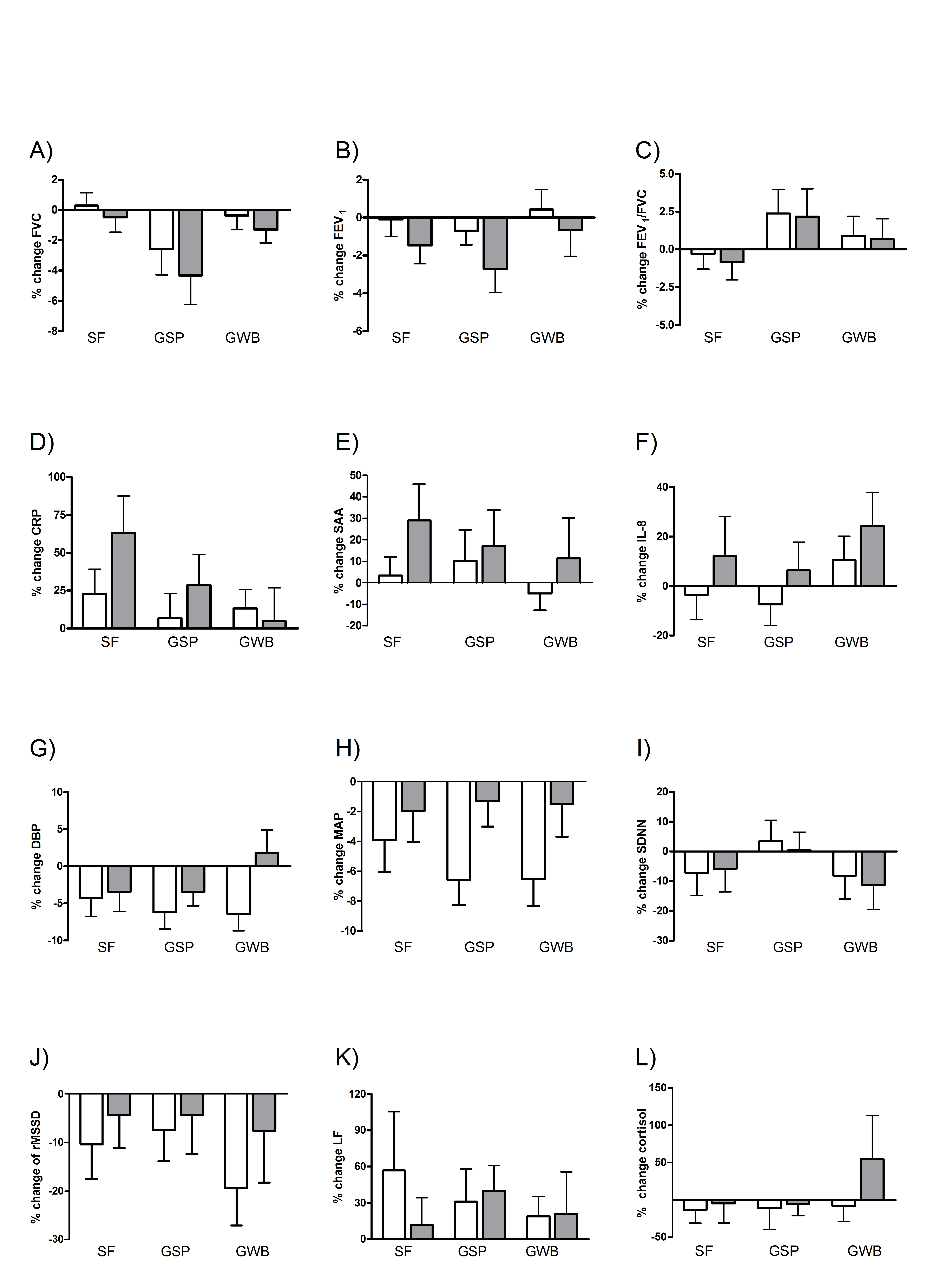

Supplement: Additional file 2: — Percent changes in A) FVC, B) FEV 1 , C) FEV 1 /FVC, D) CRP, E) SAA, F) IL-8, G) DBP, H) MAP, I) SDNN, J) rMSSD, K) LF, and L) cortisol at SF, GSP, and GWB for post- and 24 h measurements, compared to pre-exposure measurements. Health outcomes were analyzed using a repeated-measures ANOVA to assess statistical significance between locations, followed by a Student Newman-Keul’s post hoc test. Values represent mean ± SE. *p value < 0.05. [file 12940_2015_49_MOESM2_ESM.jpeg]
